# Early initiation of breastfeeding and severe illness in the early newborn period: An observational study in rural Bangladesh

**DOI:** 10.1371/journal.pmed.1002904

**Published:** 2019-08-30

**Authors:** Shahreen Raihana, Michael J. Dibley, Mohammad Masudur Rahman, Tazeen Tahsina, Md. Abu Bakkar Siddique, Qazi Sadequr Rahman, Sajia Islam, Ashraful Alam, Patrick J. Kelly, Shams El Arifeen, Tanvir M Huda

**Affiliations:** 1 The University of Sydney, Faculty of Medicine and Health, Sydney School of Public Health, New South Wales, Australia; 2 Maternal and Child Health Division, International Centre for Diarrhoeal Disease Research, Bangladesh (icddr,b), Dhaka, Bangladesh; 3 Department of Health Promotion, Education, & Behavior, Norman J Arnold School of Public Health, University of South Carolina, Columbia, United States of America; Cornell University, UNITED STATES

## Abstract

**Background:**

In Bangladesh, neonatal sepsis is the cause of 24% of neonatal deaths, over 65% of which occur in the early-newborn stage (0–6 days). Only 50% of newborns in Bangladesh initiated breastfeeding within 1 hour of birth. The mechanism by which early initiation of breastfeeding reduces neonatal deaths is unclear, although the most likely pathway is by decreasing severe illnesses leading to sepsis. This study explores the effect of breastfeeding initiation time on early newborn danger signs and severe illness.

**Methods and findings:**

We used data from a community-based trial in Bangladesh in which we enrolled pregnant women from 2013 through 2015 covering 30,646 newborns. Severe illness was defined using newborn danger signs reported by The Young Infants Clinical Science Study Group. We categorized the timing of initiation as within 1 hour, 1 to 24 hours, 24 to 48 hours, ≥48 hours of birth, and never breastfed. The analysis includes descriptive statistics, risk attribution, and multivariable mixed-effects logistic regression while adjusting for the clustering effects of the trial design, and maternal/infant characteristics. In total, 29,873 live births had information on breastfeeding among whom 19,914 (66.7%) initiated within 1 hour of birth, and 4,437 (14.8%) neonates had a severe illness by the seventh day after birth. The mean time to initiation was 3.8 hours (SD 16.6 hours). The proportion of children with severe illness increased as the delay in initiation increased from 1 hour (12.0%), 24 hours (15.7%), 48 hours (27.7%), and more than 48 hours (36.7%) after birth. These observations would correspond to a possible reduction by 15.9% (95% CI 13.2–25.9, *p* < 0.001) of severe illness in a real world population in which all newborns had breastfeeding initiated within 1 hour of birth. Children who initiated after 48 hours (odds ratio [OR] 4.13, 95% CI 3.48–4.89, *p* < 0.001) and children who never initiated (OR 4.77, 95% CI 3.52–6.47, *p* < 0.001) had the highest odds of having severe illness. The main limitation of this study is the potential for misclassification because of using mothers’ report of newborn danger signs. There could be a potential for recall bias for mothers of newborns who died after being born alive.

**Conclusions:**

Breastfeeding initiation within the first hour of birth is significantly associated with severe illness in the early newborn period. Interventions to promote early breastfeeding initiation should be tailored for populations in which newborns are delivered at home by unskilled attendants, the rate of low birth weight (LBW) is high, and postnatal care is limited.

**Trial registration:**

Trial Registration number: anzctr.org.au ID ACTRN12612000588897.

## Abstract 2

Shahreen Raihana and colleagues reveal the importance of early initiation of breastfeeding to prevent against illness in newborns in Bangladesh.

## Author summary

**Why was this study done?:**

- Severe illness, including sepsis, is one of the leading causes of newborn deaths in low-and-middle-income countries and is responsible for 42% deaths in the early neonatal period (0–7 days).
- WHO currently stresses the importance of initiating breastfeeding within the first hour of birth.
- We identified studies conducted in Egypt, India, Nepal, Ghana, Tanzania, and Ethiopia in the past 2 decades that specifically examined the role of timing of breastfeeding initiation and neonatal survival.
- We found no direct evidence reporting the mechanism by which initiation within the first hour of birth can reduce early newborn (0–7 days) deaths.

**What did the researchers do and find?:**

- We report the effect of early initiation of breastfeeding on severe illnesses in the early newborn period using data from a large population-based cohort.
- We defined severe illness using newborn danger signs reported in The Young Infants Clinical Signs Study Group.
- Our results show that the earlier the initiation of breastfeeding, the lower the risk of having severe illnesses in the early newborn stage.
- By accounting for possible reverse causality from infants too ill to initiate breastfeeding, we have established that starting breastfeeding beyond the first hour of life can double the likelihood of having severe illness.

**What do these findings mean?:**

- Early initiation of breastfeeding within 1 hour of birth reduces neonatal mortality, and a reduction in the rate of severe illnesses, including suspected sepsis, likely mediates this effect.
- Our work highlights the need to design and evaluate interventions to promote and support early initiation of breastfeeding that engage women who are at the highest risk of delaying initiation of breastfeeding, as well as those assisting them at delivery.
- Policymakers need to prioritize the effective implementation of interventions to support early and sustained exclusive breastfeeding as priorities for global public health.

## Introduction

Globally 2.7 million newborns died during the first 28 days of life (0–27 days) in 2015 [1]. More than a third of these neonatal deaths occurred on the first day, and three-quarters in the early neonatal period, i.e., 0 to 6 days [2,3]. In Bangladesh, overall neonatal mortality has decreased from 55 per 1,000 live births in 1990 [4] to 23 per 1,000 live births in 2015 [1], with an average yearly reduction of 1 death per 1,000. However, this reduction is 3 times slower than the reduction of under-5 mortality over the same period, and neonatal deaths now account for 62% of all under-5 deaths [1], whereas early-neonatal deaths (19.3/1,000) contribute to over 65% of all neonatal deaths.

Sepsis is considered the final common pathway to neonatal death due to severe illnesses and various invasive infections [5]. Neonatal sepsis is one of the leading causes of neonatal deaths in developing countries and is responsible for 13% of deaths in the neonatal period and 42% of deaths in the first 7 days of life [6]. A quarter of the neonatal deaths in Bangladesh are a result of severe illnesses, including, sepsis, tetanus, and diarrhea [1,3]. Despite being highly preventable, severe illnesses including sepsis is a predominant cause of death among newborns globally [7,8] and in Bangladesh [9].

WHO recommends early initiation of breastfeeding within 1 hour of birth as the first step toward ensuring optimal breastfeeding [10]. Literature [11,12] suggests that successful early initiation of breastfeeding facilitates sustained optimal breastfeeding practices throughout infancy. Yet only about 2 in every 5 newborns worldwide and 40% of newborns in South Asia begin breastfeeding within the first hour of life [10,12,13]. In Bangladesh, although there have been some improvements in other breastfeeding practices, including exclusivity and duration of breastfeeding, 49% of children are still initiating breastfeeding after the first hour of birth [14,15].

Recent systematic reviews [16,17] and global reports by WHO [13,18] have revealed that the risk of neonatal mortality increases by about 33% with the delay in initiation between 2 to 23 hours compared with those who initiated within the first hour of birth. Studies from Nepal, Ghana, and India [11,19,20] have reported a protective effect of early initiation of breastfeeding with a 44% reduction in the risk of death among babies surviving the first 48 hours and a 42% reduction in the risk of death among low birth weight (LBW) babies. Timely initiation of breastfeeding can be crucial in reducing infection associated with neonatal mortality [16,20] by enhancing the newborns’ immune response to infectious pathogens [21,22]. It is still not confirmed whether early initiation of breastfeeding leading to a lower incidence of severe illnesses leading to suspected sepsis is the most likely pathway to reduce neonatal deaths, especially in the first 7 days of life. There exists a gap in the evidence explaining the mechanism by which delayed initiation of breastfeeding may impact mortality in resource-limited settings of developing countries. Early initiation ensures that the infant receives colostrum, which is rich in several immunoglobulins and nutritional factors protecting the newborn from short- and long-term illnesses and death [20,23].

Although several articles report the importance of exclusive breastfeeding and duration of breastfeeding in infancy [24–26], we found no study that looked at the influence of the time of initiation of breastfeeding on newborn danger signs with an emphasis on severe illnesses in the early newborn period, i.e., in the first 7 days. In this paper, we aim to explore the effect of timing of breastfeeding initiation on severe illnesses and newborn danger signs among newborns in the first 7 days after birth in rural Bangladesh.

## Methods

### Parent trial and settings

We collected data for this analysis from a large community-based randomized controlled trial of the effect of iron folic-acid supplementation started early (≤12 weeks) and sustained throughout pregnancy on neonatal mortality. Details of the methods and design of the trial are published as a study protocol by Huda and colleagues [27]. We conducted the trial between 2013 and 2015 in the rural areas of 5 districts in Bangladesh. All women aged between 15 and 49 years at the time of enrollment are permanent residents of the study area and became pregnant during the enrolment period were recruited as study participants. These women were followed throughout pregnancy until 6 weeks after pregnancy outcome. Pregnant women were identified by study personnel from active pregnancy surveillance conducted by BRAC (an international nongovernment organization) field workers (*Shyastho Sebikas*) to identify women with menses delayed more than 45 days from the first date of their last menstrual cycle. Study personnel then enrolled consenting women following confirmation of pregnancy using a “pregnancy confirmatory dipstick test.” The analysis for this paper was planned at the time the trial was set up, and the questions required for this analysis was designed before the trial was implemented. We registered the trial with the Australia New Zealand Clinical Trials Registry (ANZCTR). The registration ID is ACTRN12612000588897.

### Data collection

Enrollment, follow-up, and data collection was identical throughout the study area. We collected follow-up information from each woman until 42 days after pregnancy outcome. Baseline data including demographic and socioeconomic indicators, intake of nutritional supplements (prior to enrollment and during the study period), parental education, birth history, and maternal health condition during pregnancy. Incentives were given to enrolled women for notifying the research team of the birth within 24 hours of delivery. Study personnel collected information at 48 hours after birth on essential newborn care practices and at the 7 to 10 day follow-up visit on day-to-day neonatal danger signs from birth until the seventh day after birth.

On the first postbirth visit (at 48 hours of birth), mothers were asked if they had ever breastfed their newborn, and if they did, how much time elapsed between birth and initiation of breastfeeding. In case a live-born infant died before the first postbirth follow-up visit, we still collected information on breastfeeding initiation for the child before their death. We also measured the birth weight of the child at this visit using a portable digital scale and infant weighing pouch (WeiHeng WH-A08). At the 7 to 10 day follow-up visit, we asked the mothers to give a day-by-day account of the onset, continuation, and cessation of each of the danger signs during the first 7 days. For infants who died within 7 days of birth, we collected the danger signs before death and included them in the analysis. We estimated the gestational age of the infants in weeks, from the last menstrual period reported by mother at enrollment until the reported date of birth outcome.

### Study definitions

We included in the analysis live-born infants with known breastfeeding initiation status, including those who were never breastfed. Breastfeeding initiation was the time in hours after birth, when the newborn was first put to the mother’s breast. To estimate the extent of adherence and nonadherence to WHO recommendation of initiation within an hour of birth, we categorized the variable as within 1 hour, ≥1 hour to <24 hours, ≥24 hours to <48 hours, ≥48 hours, and never breastfed. We excluded women whose breastfeeding status was unknown or missing.

The outcome variable severe illness was defined using newborn danger signs reported in The Young Infants Clinical Signs Study Group [28,29] and the Bangladesh Neonatal Health Strategy [30]. We identified severe illness in newborns if their mother reported the presence of any 1 of the following 6 signs and symptoms: unusually cold/clammy skin, high body temperature, unconscious/no movement or lethargic, caregivers report of convulsions, rapid breathing or difficulty in breathing, and unable to breastfeed. We did not have information on the seventh sign used in previous studies, namely, severe chest indrawing. Use of this definition for severe illness is recommended for studies in low resource settings, in which suspected sepsis cannot be confirmed with the usual clinical parameters and cut-offs [31,32]. We present severe illness as a binary outcome variable indicating presence or absence of the outcome of interest.

Potential confounders were classified as infant, maternal, and household characteristics. Infant characteristics included the sex of the child (male and female); the birth weight [≥2,500 g, 2000–2499 g (LBW), and ≤2000 g (LBW)]; the birth outcome [multiple and single]; colostrum (given and not given); appropriate care of the umbilical cord; application of material after cutting the umbilical cord; timing of the first bath after birth (within and after 72 hours); and timing of drying after birth (within and after 5 minutes). Maternal characteristics included age (<20 years and ≥20 years); gestational age at birth (<28 weeks, 28–33 weeks, 34–36 weeks, and ≥37 weeks); education (no education, primary completed, secondary completed, and higher); parity (primiparous and multiparous); stillbirth/miscarriage of previous child; prolonged labor during childbirth; and fever during childbirth. In addition, we constructed a combined variable for type, place, and skilled attendance at delivery (normal vaginal delivery [NVD] at facility by skilled attendant, NVD at home by unskilled attendant, NVD at home by skilled attendant, and caesarean section (CS) at facility by skilled attendant). Household characteristics included the wealth quintiles. We used standard demographic health survey methods to form a list of household assets to construct the wealth index [33]. Most of the selected potential confounders were reported in previous studies that explored the determinants of the timing of initiation of breastfeeding [11,19,20,22,23].

### Statistical analysis

We used frequency distributions to describe the baseline characteristics of the study population and summarized all data on household, maternal, and infant characteristics using descriptive statistics of proportions for categorical variables. All descriptive statistics are presented by the early onset of newborn danger signs for severe illnesses in the newborns. The mean time to initiate breastfeeding is calculated among children for whom we have a record of their breastfeeding initiation time.

To examine the effect of time of initiation of breastfeeding on the primary outcome of severe illnesses in the early neonatal period, we used a multivariable mixed-effects logistic regression model, adjusting for infant, maternal, and household characteristics that showed an independent association (*p* < 0.20) with the outcome in an univariable model. The variance inflation factor was used to assess collinearity between covariates included in the multivariable model. We examined the outcome variable and found it varied by cluster (data not shown) indicating the need to use multilevel mixed-effects models. All regression models were adjusted for clustering of participants from 100 clusters (from a cluster randomized control trial [RCT]) in 5 districts using multilevel random effects, with clusters nested within district [34]. The variable for treatment arms had been included in all regression models in order to account for the effects of the intervention. We present the odds ratios (ORs) and 95% CIs for severe illness for each category of time to initiate breastfeeding.

To examine the probability of potential reverse causality due to maternal infections and complications at childbirth, we performed a sensitivity analysis excluding the infants whose mother reported prolonged labor or fever around the time of childbirth. We then fitted a restricted version of the adjusted model (restricted model 1) for the same covariates. Further, to explore the probability of reverse causality due to the child being too ill to initiate breastfeeding early, we performed another sensitivity analysis, excluding the children who died in the first 48 hours of birth. We fitted this restricted model (restricted model 2), adjusting for the same covariates of the adjusted model. Finally, we compared the adjusted OR (aOR) of all 3 models to identify any possible influence of reverse causality due to maternal complications during the time of delivery or severe illness of the child immediately after birth.

The population attributable fraction (PAF) of severe illnesses among newborns whose breastfeeding was initiated 1 hour, 24 hours, or 48 hours of birth were calculated using the formula below [35,36]:

$$PAF=P_{b}(\frac{OR-1}{OR}).$$

Here, *P*_*b*_ is the proportion of children with breastfeeding initiated after 1 hour, 24 hours, or 48 hours of birth, and OR is the OR of initiating breastfeeding after 1 hour, 24 hours, and 48 hours of birth. For ease of interpretation, we will present the PAF as population attributable risk percent (PAR%) by multiplying PAF by 100. We calculated the crude PAR% using the ORs from the univariable association. To measure the adjusted contribution of delayed initiation of breastfeeding on the total risk of having severe illness, the adjusted PAR% was calculated using the aORs from both the final adjusted model and restricted model. We estimated 95% CI for all PAR%. We performed all statistical analyses using STATA version 15 (Stata Corporation, College Station, TX).

### Ethics

The Ethical Review Committee (ERC) of the International Centre for Diarrhoeal Disease Research, Bangladesh (icddr,b), and the Human Research Ethics Committee (HREC) of the University of Sydney have granted ethics approval for the parent study. We obtained written informed consent from pregnant women during enrollment into the study, which provided full disclosure regarding the study.

## Results

**Fig 1:**
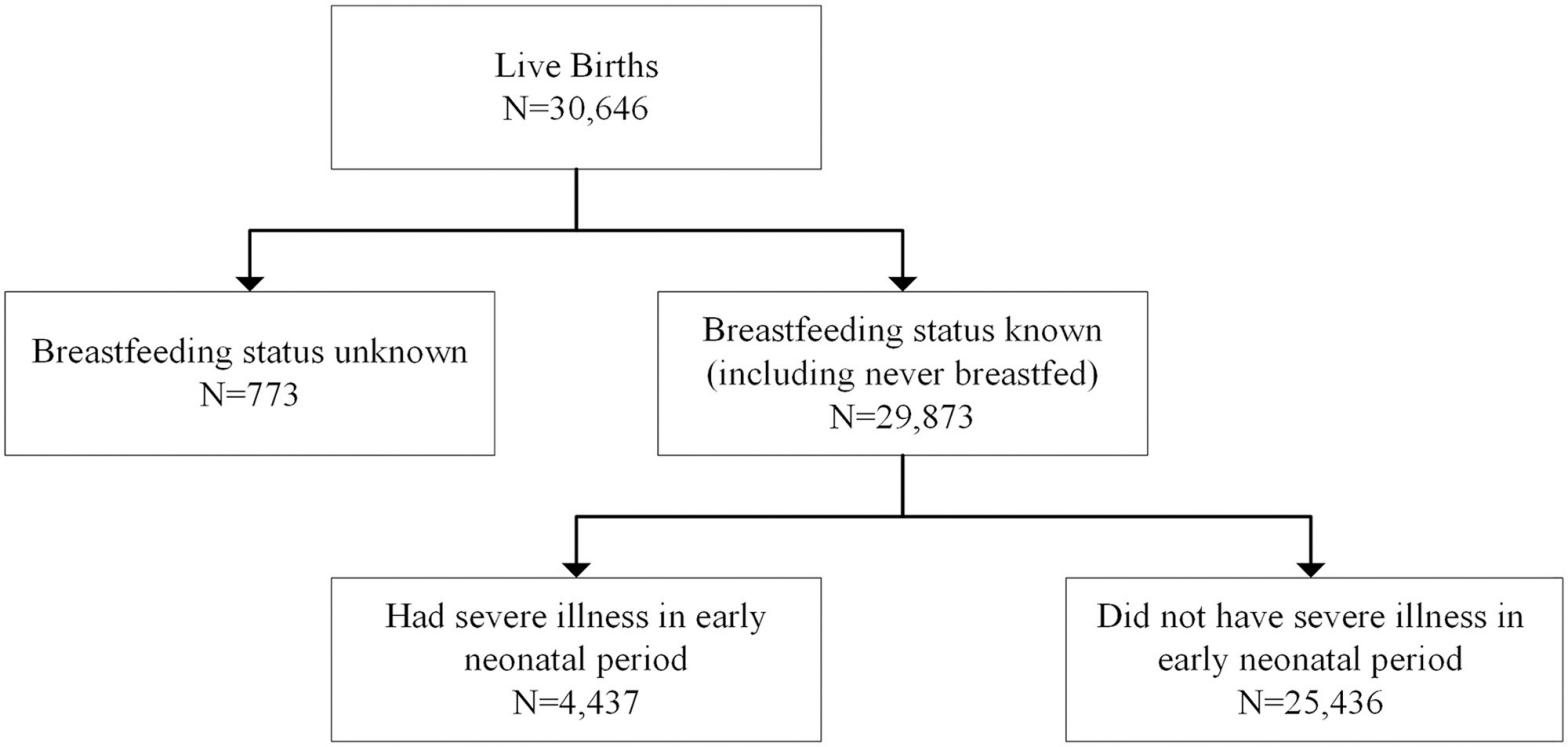
Flowchart of infants with defined breastfeeding initiation time, included in the analysis.

**Fig 2:**
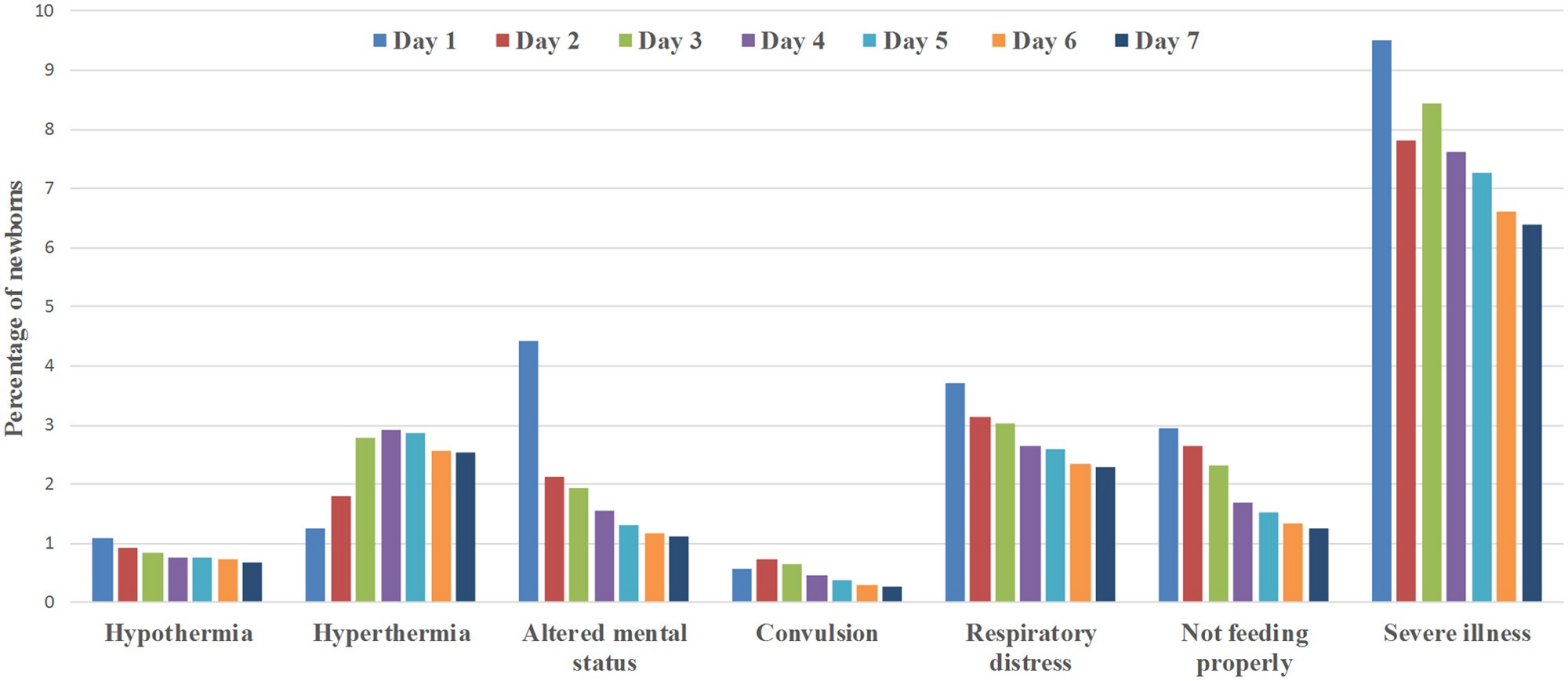
Percentage of newborns (*N* = 29,873) presenting with any danger signs during the first 7 days of birth.

There were 30,646 live-born infants during the study period, and time to initiation of breastfeeding was known for 29,873 (97.5%) infants. The remaining 773 women had no information on their breastfeeding status, and we excluded them from the analysis. Two-thirds of the newborns (66.7%) with known breastfeeding initiation status had initiated breastfeeding within 1 hour of birth. The mean time to initiate breastfeeding, among children with a record of their breastfeeding initiation time was, 3.8 hours (SD 16.6 hours). By the end of 48 hours, all but 5.7% of infants were breastfed. Severe illness in the neonatal period early (0–6 days) was reported for 4,437 (14.9%; Fig 1) infants of whom 338 (7.6%) subsequently died within the first 7 days. Among the severe illness cases, hyperthermia was the most common danger sign reported by 36.6% mothers, followed by respiratory distress reported by 37.4% mothers. Convulsion was the least reported danger sign and was reported by 7.8% mothers. Fig 2 shows the percentages of newborns who had any of the danger signs in the first 7 days after birth. Table 1 summarizes some infant, maternal, and household characteristics of the newborns by their time of breastfeeding initiation.

**Table 1:** Distribution of infant, maternal, and household characteristics of study participants by time when breastfeeding was initiated.

Characteristics	Breastfeeding initiated	
	Within 1 hour (*n* = 19,914)	After 1 hour (*n* = 9,959)
**Infant characteristics**		
**Sex of child**		
Female	9,461 (47.5)	4,740 (47.6)
Male	10,453 (52.5)	5,219 (52.4)
**Birth weight**		
≥2,500 g	13,917 (76.7)	7,025 (78.4)
2,000–2,499 g	3,571 (19.7)	1,495 (16.7)
<2,000 g	660 (3.6)	438 (4.9)
**Birth outcome**		
Single	19,773 (99.3)	9,732 (97.7)
Multiple	141 (0.7)	227 (2.3)
**Colostrum**		
Given	19,757 (99.2)	8,826 (88.6)
Not given	157 (0.8)	1,133 (11.4)
**Instrument boiled before the cord was cut**		
Boiled	16,386 (82.3)	7,719 (77.5)
Not boiled	3,528 (17.7)	2,240 (22.5)
**Application of material after cutting cord**		
Applied nothing	17,298 (86.9)	7,919 (79.5)
Material applied to cord	2,616 (13.1)	2,040 (20.5)
**Time of first bath**		
After 72 hours	11,452 (57.5)	6,204 (62.3)
Within 72 hours	8,462 (42.5)	3,755 (37.7)
**Timing of drying**		
Within 5 minutes	11,730 (58.9)	4,552 (45.7)
After 5 minutes/not dried	8,184 (41.1)	5,407 (54.3)
**Maternal Characteristics**		
**Mother's age**		
≥20 years	15,817 (79.4)	7,617 (76.5)
<20 years	4,097 (20.6)	2,342 (23.5)
**Gestational age at birth**		
≥37 weeks	14,647 (73.6)	7,177 (72.1)
34–36 weeks	3,163 (15.9)	1,650 (16.6)
28–33 weeks	1,858 (9.3)	997 (10.0)
<28 weeks	246 (1.2)	135 (1.4)
**Maternal education**		
Higher	1,026 (5.2)	606 (6.1)
Secondary	7,827 (39.3)	4,360 (43.8)
Primary	6,195 (31.1)	2,937 (29.5)
No education	4,866 (24.4)	2,056 (20.6)
**Parity**		
Multiparous	13,070 (65.6)	6,118 (61.4)
Primiparous	6,844 (34.4)	3,841 (38.6)
**Type, place, and skilled attendance at delivery**		
NVD facility skilled	2,357 (11.8)	1,334 (13.4)
NVD home unskilled	14,096 (70.8)	5,394 (54.2)
NVD home skilled	1,230 (6.2)	457 (4.6)
CS facility skilled	2,231 (11.2)	2,774 (27.9)
**Stillbirth/miscarriage of previous child**		
No	19,012 (95.5)	9,478 (95.2)
Yes	902 (4.5)	481 (4.8)
**Prolonged labor during childbirth**		
No	16,221 (81.5)	7,602 (76.3)
Yes	3,693 (18.5)	2,357 (23.7)
**Fever (mother) at childbirth**		
No	19,636 (98.6)	9,735 (97.8)
Yes	278 (1.4)	224 (2.2)
**Household characteristics**		
**Asset**		
First (Lowest)	4,101 (20.6)	1,904 (19.1)
Second	4,157 (20.9)	1,794 (18.0)
Middle	4,276 (21.5)	1,722 (17.3)
Fourth	4,007 (20.1)	2,100 (21.1)
Fifth (Highest)	3,373 (16.9)	2,439 (24.5)

**Abbreviations**: CS, caesarean section; NVD, normal vaginal delivery

Table 2 presents the unadjusted ORs and aORs for the association between timing of initiation of breastfeeding and severe illness in early neonatal stage using univariable and multivariable models from the unrestricted and restricted data. There is a dose response of higher likelihood of severe illness with an increased delay in breastfeeding initiation. Infants who initiated breastfeeding between 1 to 23 hours of birth had significantly higher odds (unadjusted OR 1.45, 95% CI 1.33–1.58) of having signs of severe illness compared with children who initiated breastfeeding within 1 hour of birth. This effect was further seen to have increased for children who initiated between 24 to 47 hours (unadjusted OR 2.94, 95% CI 2.42–3.58) and after 48 hours (unadjusted OR 4.96, 95% CI 4.29–5.73).

**Table 2:** Unadjusted OR and aOR of presenting with any one early newborn danger signs (severe illness) among newborns (0–6 days) by breastfeeding initiation time categories (*N* = 29,873).

Breastfeeding initiation time	Breastfeeding initiated *n* (%)	Severe illness *n* (%)	Unadjusted OR (95% CI)	aOR^†^ (95% CI)	aOR^††^ (95% CI) using RD1^a^	aOR^†^ (95% CI) using RD2^b^
<1 hour	19,914 (66.7)	2,382 (12.0)	1.00	1.00	1.00	1.00
1 to <24 hours	7,644 (25.6)	1,200 (15.7)	1.45 (1.33–1.58)*	1.37 (1.25–1.50)*	1.41 (1.29–1.55)*	1.36 (1.24–1.49)*
24 to <48 hours	599 (2.0)	166 (27.7)	2.94 (2.42–3.58)*	2.85 (2.31–3.52)*	2.87 (2.32–3.55)*	2.87 (2.32–3.54)*
≥48 hours	1,016 (3.4)	373 (36.7)	4.96 (4.29–5.73)*	4.13 (3.48–4.89)*	4.26 (3.59–5.04)*	4.15 (3.49–4.92)*
Never breastfed	700 (2.3)	316 (45.1)	7.05 (5.99–8.31)*	4.77 (3.52–6.47)*	5.06 (3.74–6.85)*	5.96 (4.29–8.28)*

^†^Adjusted for sex of child; birth weight; instrument boiled before the cord was cut; application of material after cutting cord; time of first bath; timing of drying; colostrum; gestational age at birth; parity; type, place, and skilled attendance at delivery; stillbirth/miscarriage of previous child; prolonged labour during childbirth; fever (mother) at childbirth; assets; and the treatment arms. Also adjusted for clustering of participants from 100 clusters in 5 districts using multilevel random effects (clusters nested within district).

^††^Adjusted for all the above mentioned variables except prolonged labor during childbirth and fever (mother) at childbirth. Also adjusted for clustering effect.

^a^Excluding children (*n* = 6,340) whose mother reported maternal complications (prolonged labor or fever) during the time of delivery.

^b^Excluding children (*n* = 470) who died in the first 48 hours.

*p < 0.001

**Abbreviations**: aOR, adjusted odds ratio; OR, odds ratio; RD, restricted data

When compared with newborns with early initiation of breastfeeding, the aORs for severe illness (Table 2) remained significantly higher among all late breastfeeding initiator groups (1–23 hours: aOR 1.37, 95% CI 1.25–1.50; 24–47 hours: aOR 2.85, 95% CI 2.31–3.52; and 48 hours or more: aOR 4.13, 95% CI 3.48–4.89). The highest risk for severe illness and early newborn danger signs was for those who were unable to initiate breastfeeding (aOR 4.77, 95% CI 3.52–6.47) within the 7 days. The multivariable models were adjusted for confounders that had univariable *p* < 0.2 (S1 Table). All variance inflation factors were <5, indicating that collinearity was not an issue for the multivariable models.

In the adjusted model using unrestricted data (S1 Table), the odds of having severe illness was lower for mothers who delivered their child by unskilled birth attendants at home (aOR 0.75, 95% CI 0.67–0.84), compared with women who delivered at a facility with skilled attendants. Performing CS at a facility by skilled attendants was associated with a reduced risk of early newborn danger signs (aOR 0.60, 95% CI 0.52–0.68). Infants born with a birthweight of ≥2,500g and who received appropriate thermal and cord care at birth had significantly (p<0.0001) lower odds of having severe illnesses than those who had LBW and inappropriate postbirth care. Infants who were first born, whose mother’s preceding pregnancy ended in stillbirth or miscarriage, and whose mother has prolonged labor during childbirth had significantly higher odds of having severe illnesses in the first 7 days after birth.

Sensitivity analysis of the adjusted association after excluding the children whose mother had prolonged labor or fever at childbirth (Table 2) resulted in slightly higher odds of severe illness among all the breastfeeding initiation categories. The aORs of having early newborn danger signs and severe illness among late initiators slightly increased for children of women who did not have prolonged labor or fever during childbirth. About one-fifth (18.5%) of the 19,914 women who initiated within the first hour had prolonged labor. The increased likelihood of early newborn danger signs in the reference group leads to an overall increase in odds of severe illnesses among all late initiators. Excluding children who died in the first 48 hours (Table 2) had little to no effect on the adjusted risk of early newborn danger signs and severe illnesses among late initiators compared with the final adjusted model. The unadjusted and adjusted associations between severe illnesses in the early neonatal stage and a range of confounders used to generate the models using unrestricted and restricted data are shown in S1 Table. The association between breastfeeding initiation time and severe illness were similar when the regression model was adjusted for all covariates as continuous variables (S2 Table).

Table 3 shows that the PAR% decreases with increased delay in breastfeeding initiation. After adjusting for confounders, about 16% of the early newborn danger signs and severe illness could have been reduced if all newborns initiated breastfeeding within the first hour of birth. The PAR% for initiating breastfeeding after 24 hours indicates that 10.6% of the total risk of early newborn danger signs and severe illnesses were attributable to initiating breastfeeding after the first day of birth.

**Table 3:** Crude and adjusted PAR% of severe illness in all breastfeeding initiation time categories.

Breastfeeding initiation time	Crude PAR% (95% CI)	Adjusted PAR% (95% CI)	Adjusted PAR% (95% CI) RD1^a^	Adjusted PAR% (95% CI) RD2^b^
After 1 hour	23.6 (21.1–25.9)	15.9 (13.2–18.5)	16.7 (14.0–19.4)	16.0 (13.2–18.7)
After 24 hours	14.0 (12.7–15.4)	10.6 (9.1–12.0)	10.8 (9.3–12.3)	10.7 (9.1–12.2)
After 48 hours	11.9 (10.6–13.1)	8.5 (7.2–9.8)	8.8 (7.5–10.1)	8.5 (7.1–10.0)

^a^Excluding children (*n* = 6,340) whose mother reported maternal complications (prolonged labor or fever) during the time of delivery.

^b^Excluding children (*n* = 470) who died in the first 48 hours.

**Abbreivations**: PAR%, population attributable risk percent; RD, restricted data

## Discussion

We found that early initiation of breastfeeding within an hour of birth was significantly associated with a reduced risk of early newborn (0–6 days) danger signs and severe illnesses in rural Bangladesh. We detected a dose-response relationship of increasing odds of severe illnesses in the early newborn stage with greater delay in initiation of breastfeeding after adjusting for confounders. Our findings indicate that the highest risk of presenting with early newborn danger signs following delayed initiation of breastfeeding beyond the first hour was with newborns who were first born, had very LBW (<2,000 g), did not receive appropriate cord and thermal care, were born through NVD at home with unskilled attendance, had a history of stillbirth/miscarriage of a previous sibling, or whose mother had prolonged labor during childbirth. We also found that one-sixth of the reported danger signs in the early neonatal period could be reduced if children who initiated beyond the first hour could initiate within the recommended time. Excluding children whose mother had maternal complications and those who died within 48 hours had a minimum to no effect on the fraction of severe illness cases that could be prevented. The findings from this study highlight the need to encourage women and caregivers to facilitate early initiation of breastfeeding to reduce early newborn danger signs and severe illnesses, especially among high-risk newborns.

Our study is one of the few studies that specifically explores the effect of delayed breastfeeding initiation on severe illness during the early newborn stage (0–6 days after birth), the most crucial stage of the neonatal period [37]. Most studies on the health effects of early initiation of breastfeeding have examined all-cause neonatal mortality [11,19,20,23] and infectious diseases related to neonatal mortality such as diarrhea and respiratory infections [16,38] during the entire neonatal period. In this study, information on the timing of initiation and danger signs was collected within 7 to 10 days of birth, thus reducing the recall period of reporting both the exposure and outcome. As the study sample originates from a large community-based randomized controlled trial, we were able to collect data from a large number of newborns. Sensitivity analyses excluding infants who died within 48 hours of birth, as done in several previous studies looking at mortality outcomes [11,19,20,23], resulted in no change in the risk of severe illnesses in relation to the time of initiation of breastfeeding. Even though our analysis does not provide direct evidence of the absence of reverse causation, it is very unlikely that the reported danger signs were due to the newborn being too sick to initiate breastfeeding within 1 hour of birth. Further sensitivity analysis indicates that maternal complications and signs of maternal infections around the time of childbirth may have contributed to a higher risk of severe illnesses among the newborns in all categories of timing of initiation of breastfeeding, including those who initiated within an hour of birth. The study included all newborns from a defined area of rural Bangladesh over a 12-month recruitment period and thus increased the generalizability of the study results. We present the dose-response relationship of early initiation of breastfeeding associated with a reduced risk of severe illnesses in the early neonatal period after adjusting for all possible confounders known to be associated with the timing of initiation of breastfeeding and early newborn danger signs.

Our study has several limitations. Firstly, the danger signs for severe illnesses used in this study includes 6 out of the 7 signs and symptoms proposed in the Young Infants Clinical Signs Study [28], but we did not collect the seventh danger sign, severe chest indrawing. A study in Bangladesh [39] found that severe chest indrawing has very low specificity and thus contributes to overestimating the proportion of children with severe illness requiring hospital admission. Thus, omitting this symptom in the outcome in our study will not lead to an overestimate of the effect of breastfeeding on newborn danger signs. In addition, there exists a potential for misclassification of the outcome considering that severe illness was defined using the mother’s report of episodes of danger signs, and there were no confirmatory tests using biomarkers to classify the severity. Secondly, mothers of dead infants may have recalled danger signs and breastfeeding initiation times differently compared with the mothers of surviving newborns, thus incorporating a potential recall bias. The short recall period of 7 to 10 days is likely to have minimized any recall bias but not eliminated it. However, there was no change in risk of presenting with newborn danger signs following delayed initiation of breastfeeding when the model was applied to children who survived 48 hours from birth. This implies that any recall bias was minimal and provides evidence that our results were not the result of reverse causality. Considering the short recall period for all mothers, the generic approach to defining severe illnesses, and the lack of evidence of reverse causality our findings provide strong evidence that delayed initiation of breastfeeding increases the risks of early newborn danger signs and severe illness.

Timely initiation of breastfeeding is the first step toward ensuring exclusive breastfeeding and sustaining optimal breastfeeding practices [40]. Furthermore, early initiation and early attachment to the mother’s breast promotes thermal care of the infant and can reduce the risk of hypothermia immediately after birth [11]. Delayed initiation of breastfeeding has a strong biological plausibility of leading to severe illnesses, considering the important role of breastfeeding in enhancing immune functions during the early stages of life [10,41]. Delayed initiation is known to be further compounded with an early introduction of prelacteal feeding [23,40], which is known to have a detrimental effect on the immune system and has the potential to lead to infection and suspected sepsis [42]. Early exposure to breastmilk ensures intake of colostrum, which contains a high concentration of lactoferrin, immunoglobulin A (IgA), leukocytes, and specific developmental factors [43,44]. The amount of these proteins and immunoglobulin (especially IgA) [45] is significantly higher in colostrum than in the transitional milk (6 to 14 days of lactation), and mature milk (after the 15th day of lactation) [46]. Colostrum intake accelerates intestinal maturation, increases the integrity of the epithelial layer, promotes epithelial recovery from infections, has anti-inflammatory and antimicrobial potential, and decreases the risk of microbial translocation [41,47].

Findings in this study are consistent with previous studies that looked at the relationship between early initiation of breastfeeding and all-cause mortality [11,19,20,23] and morbidity [16,38]. Studies in Ghana [11], India [20], Nepal [19], Egypt [22], and Tanzania [47] report a similar dose-response relationship of early initiation of breastfeeding on survival. The rate of initiation of breastfeeding within the first hour is higher in this study than that reported in studies from Nepal (3.4%) [19] and Ghana (43%) [11] and lower than reported in studies from Tanzania (87%) [47]. The study from India [20] looked at the change in risk of adverse outcomes associated with initiation within the first day and later, rather than the association for initiation within the first hour. The rates of LBW reported in studies from Nepal (29.8%) [19] and India (22%) [20] were similar to our study and both present the higher risk of delayed initiation of breastfeeding associated with adverse outcomes among LBW infants (<2,500 g).

In this study, we found that prolonged labor and fever during childbirth contributed to a higher risk of severe illnesses in all breastfeeding initiation groups, especially in the reference group of those who initiated within 1 hour of birth, because two-thirds of the children fall in this group. A higher proportion of mother-infant dyads who never initiated breastfeeding reported having prolonged labor compared with newborns who initiated within an hour of birth (35.7% versus 18.5%). Previous studies [48–50] support these findings and provide evidence that neonatal infection in the first week of life is associated with maternal infection and intrapartum fever. Another study suggests that women who had delivery complications were less likely to initiate breastfeeding on time [51].

We found that women who had caesarean delivery at a facility by skilled attendants had a lower likelihood of presenting with early newborn danger signs in association with delayed breastfeeding initiation compared with those who had a NVD. Previous studies, mostly in high-income countries, compared NVD at a facility with caesarean delivery at a facility and showed more risk of infections in the latter group [40,52–54]. Findings in our study may have resulted from the high percentage (70%) of all births taking place at home, mostly in unhygienic and infection-prone surroundings with limited postnatal support. Hence, the adjusted association showed that babies born through caesarean delivery by skilled attendance at a facility had a lower likelihood of severe illness compared with NVD at a facility.

The findings in this study indicate that the timing of initiation of breastfeeding, especially within the first hour of birth, is significantly associated with reduced risk of severe illness in the early neonatal period. Hence the findings support the need to reinforce the implementation of existing recommendations from WHO and UNICEF [13] to “place the child in skin-to-skin contact with mothers immediately following birth for at least an hour and encourage the mother to initiate breastfeeding within this time,” as the Step 4 of the 10 steps towards successful breastfeeding [16,55]. By improving early initiation of breastfeeding, newborn morbidity and mortality would likely decrease and could contribute to achieving Sustainable Development Goals (SDG) 2030 for reducing child mortality.

Our findings indicate the need for setting a future research agenda around developing comprehensive community-based interventions to promote initiation of breastfeeding within the first hour of birth in low- and middle-income countries to reduce early newborn danger signs and severe illness and thus reduce neonatal deaths. It is crucial for healthcare providers and birth attendants to encourage and support timely initiation regardless of the mode of delivery and birth weight of the newborn [54,56]. Interventions to promote and support early breastfeeding initiation could be designed based on the place and type of delivery and requires involvement of all role players around the time of childbirth. Such role players may include family members and birth attendants (skilled or unskilled) for home deliveries, healthcare providers for NVD at a facility, and a professionally endorsed standard guideline for caesarean delivery. Interventions should be tailored to suit the needs of populations in which the rate of LBW is high and postnatal care is limited with emphasis on first born infants and mothers who experienced pregnancy complications.

## Supporting information

S1 Table Unadjusted and adjusted association of the confounders and severe illness among newborns in first 7 days of birth (*N* = 29,873). (DOCX)

S2 Table Unadjusted and adjusted odds of presenting with any one early newborn danger signs (severe illness) among newborns (0–6 days) by breastfeeding initiation time categories (*N* = 29,873) adjusted for all continuous covariates. (DOCX)

S1 STROBE Checklist STROBE, Strengthening the Reporting of Observational studies in Epidemiology. (DOC)

## Abbreviations

aOR: adjusted odds ratio
CS: caesarean section
IgA: Immunoglobulin A
LBW: low birth weight
NVD: normal vaginal delivery
OR: odds ratio
PAF: population attributable fraction
PAR%: population attributable risk percent
RCT: randomized controlled trial
RD: restricted data
SDG: Sustainable Development Goals
STROBE: Strengthening the Reporting of Observational studies in Epidemiology
