# Supplementary material for: Early initiation of breastfeeding and severe illness in the early newborn period: An observational study in rural Bangladesh
Source: PLoS Med. 2019 Aug 30;16(8):e1002904. doi: 10.1371/journal.pmed.1002904 (PMC6716628; doi:10.1371/journal.pmed.1002904)
Supplement: S1 Table — (DOCX) [file pmed.1002904.s001.docx]

**S1 Table: Unadjusted and adjusted association of the confounders and severe illness among newborns in first seven days of birth [N=29,873]**

|  | **N** | **Severe illness  n(%)** | **Unadjusted OR (95%CI)** | **Adjusted OR (95%CI)** | **Adjusted OR (95%CI) using ‘restricted data 1’ [RD1]^a^** | **Adjusted OR (95%CI) using ‘restricted data 2’ [RD2]^b^** |
| --- | --- | --- | --- | --- | --- | --- |
| Sex of child |  |  |  |  |  |  |
| Female | 14201 | 1877(13.2) | 1·00 | 1.00 | 1.00 | 1.00 |
| Male | 15672 | 2560(16.3) | 1·29 (1·21, 1·38)* | 1.3 (1.20, 1.39)* | 1.28 (1.19, 1.38)* | 1.31 (1.22, 1.41)* |
| Birthweight |  |  |  |  |  |  |
| ≥2500g | 20942 | 2885(13.8) | 1·00 | 1.00 | 1.00 | 1.00 |
| 2000-2499g | 5066 | 781(15.4) | 1·15 (1·05, 1·25)* | 1.15 (1.05, 1.26)* | 1.14 (1.04, 1.25)* | 1.15 (1.04, 1.26)* |
| <2000g | 1098 | 298(27.1) | 2·44 (2·11, 2·82)* | 1.94 (1.65, 2.27)* | 1.89 (1.61, 2.21)* | 1.91 (1.63, 2.25)* |
| Instrument boiled before the cord was cut |  |  |  |  |  |  |
| Boiled | 24105 | 3372(14.0) | 1·00 | 1.00 | 1.00 | 1.00 |
| Not boiled | 5768 | 1065(18.5) | 1·36 (1·25, 1·49)* | 1.12 (1.01, 1.23) | 1.12 (1.02, 1.24) | 1.12 (1.01, 1.23) |
| Application of material after cutting cord |  |  |  |  |  |  |
| Applied nothing | 25217 | 3533(14.0) | 1·00 | 1.00 | 1.00 | 1.00 |
| Material applied to cord | 4656 | 904(19.4) | 1·36 (1·24, 1·48)* | 1.22 (1.10, 1.34)* | 1.24 (1.12, 1.37)* | 1.22 (1.11, 1.35)* |
| Time of first bath |  |  |  |  |  |  |
| After 72 hours | 17656 | 2806(15.9) | 1·00 | 1.00 | 1.00 | 1.00 |
| within 72 hours | 12217 | 1631(13.6) | 0·72 (0·67, 0·78)* | 0.74 (0.68, 0.81)* | 0.75 (0.69, 0.82)* | 0.74 (0.68, 0.80)* |
| Timing of drying |  |  |  |  |  |  |
| Within 5 mins | 16282 | 2139(13.1) | 1·00 | 1.00 | 1.00 | 1.00 |
| After 5 min/not dried | 13591 | 2298(16.9) | 1·28 (1·19, 1·38)* | 1.24 (1.14, 1.35)* | 1.26 (1.16, 1.37)* | 1.26 (1.16, 1.37)* |
| Colostrum |  |  |  |  |  |  |
| Given | 28583 | 3946(13.8) | 1·00 | 1.00 | 1.00 | 1.00 |
| Not given | 1290 | 491(38.1) | 4·19 (3·71, 4·75)* | 1.33 (1.06, 1.67) | 1.34 (1.07, 1.68) | 1.34 (1.07, 1.68) |
| Gestational age at birth |  |  |  |  |  |  |
| ≥37 weeks | 21824 | 3030(13.9) | 1·00 | 1.00 | 1.00 | 1.00 |
| 34-36 weeks | 4813 | 788(16.4) | 1·21 (1·11, 1·33)* | 1.1 (1.00, 1.21) | 1.11 (1.01, 1.22) | 1.10 (0.99, 1.21) |
| 28-33 weeks | 2855 | 540(18.9) | 1·46 (1·31, 1·62)* | 1.18 (1.04, 1.33) | 1.18 (1.05, 1.34) | 1.18 (1.05, 1.34) |
| <28 weeks | 381 | 79(20.7) | 1·65 (1·27, 2·13)* | 1.20 (0.88, 1.64) | 1.20 (0.89, 1.64) | 1.23 (0.90, 1.68) |
| Parity |  |  |  |  |  |  |
| Multiparous | 19188 | 2677(14.0) | 1·00 | 1.00 | 1.00 | 1.00 |
| Primiparous | 10685 | 1760(16.5) | 1·28 (1·20, 1·37)* | 1.13 (1.04, 1.22)* | 1.18 (1.10, 1.28)* | 1.13 (1.05, 1.22)* |
| Type, place and skilled attendance at delivery |  |  |  |  |  |  |
| NVD^†^ Facility, Skilled attendant | 3691 | 789(21.4) | 1·00 | 1.00 | 1.00 | 1.00 |
| NVD^†^ Home, Unskilled attendant | 19490 | 2509(12.9) | 0·53 (0·48, 0·58)* | 0.75 (0.67, 0.84)* | 0.67 (0.59, 0.75)* | 0.75 (0.66, 0.84)* |
| NVD^†^ Home, Skilled attendant | 1687 | 356(21.1) | 0·85 (0·73, 0·99)* | 1.12 (0.95, 1.33)* | 1.05 (0.88, 1.24)* | 1.13 (0.95, 1.34)* |
| CS^††^ Facility, Skilled attendant | 5005 | 783(15.6) | 0·66 (0·59, 0·74)* | 0.60 (0.52, 0.68)* | 0.56 (0.49, 0.64) | 0.59 (0.51, 0.67)* |
| Stillbirth/miscarriage of previous child |  |  |  |  |  |  |
| No | 28490 | 4176(14.7) | 1·00 | 1.00 | 1.00 | 1.00 |
| Yes | 1383 | 261(18.9) | 1·21 (1·05, 1·40) | 1.21 (1.03, 1.42) | 1.21 (1.03, 1.43) | 1.23 (1.05, 1.44) |
| Prolonged labour during childbirth |  |  |  |  |  |  |
| No | 23823 | 3009(12.6) | 1·00 | 1.00 | 1.00 | 1.00 |
| Yes | 6050 | 1428(23.6) | 2·00 (1·86, 2·16)* | 1.77 (1.63, 1.92)* |  | 1.74 (1.60, 1.90)* |
| Fever (mother) at childbirth |  |  |  |  |  |  |
| No | 29371 | 4308(14.7) | 1·00 | 1.00 | 1.00 | 1.00 |
| Yes | 502 | 129(25.7) | 1·33 (1·07, 1·66) | 1.20 (0.94, 1.54) |  | 1.22 (0.95, 1.56) |
| Asset |  |  |  |  |  |  |
| Lowest | 6005 | 930(15.5) | 1·13 (1·01, 1·27) | 1.19 (1.05, 1.36) | 1.23 (1.09, 1.40)* | 1.21 (1.06, 1.37) |
| Second | 5951 | 928(15.6) | 1·19 (1·06, 1·33) | 1.20 (1.06, 1.36) | 1.23 (1.09, 1.40)* | 1.18 (1.04, 1.34) |
| Middle | 5998 | 926(15.4) | 1·19 (1·07, 1·33) | 1.18 (1.05, 1.34) | 1.22 (1.08, 1.38)* | 1.19 (1.05, 1.35) |
| Fourth | 6107 | 871(14.3) | 1·07 (0·96, 1·19) | 1.07 (0.95, 1.21) | 1.10 (0.97, 1.24)* | 1.07 (0.95, 1.21) |
| Fifth | 5812 | 782(13.5 | 1·00 | 1.00 | 1.00 | 1.00 |
| * p<0.001  ^a^ Excluding children (n=6340) whose mother reported maternal complications (prolonged labour or fever) during the time of delivery  ^b^ Excluding children (n=470) who died in the first 48 hours  † NVD- Normal Vaginal Delivery  †† CS- Caesarean Section | | | | | | |
