# Supplementary material for: Early initiation of breastfeeding and severe illness in the early newborn period: An observational study in rural Bangladesh
Source: PLoS Med. 2019 Aug 30;16(8):e1002904. doi: 10.1371/journal.pmed.1002904 (PMC6716628; doi:10.1371/journal.pmed.1002904)
Supplement: S2 Table — (DOCX) [file pmed.1002904.s002.docx]

**S2 Table: Unadjusted and adjusted odds of presenting with any one early newborn danger signs (severe illness) among newborns (0-6 days) by breastfeeding initiation time categories [N=29,873] adjusted for all continuous covariates**

|  | **Adjusted† OR (95%CI)^c^** | **Adjusted†† OR (95%CI) using ‘restricted data 1’ [RD1]^a,c^** | **Adjusted† OR (95%CI) using ‘restricted data 2’ [RD2]^b,c^** |
| --- | --- | --- | --- |
| <1 hr | 1·00 | 1·00 | 1·00 |
| 1 to <24hr | 1·37 (1·25, 1·50)* | 1·41 (1·29, 1·55)* | 1·36 (1·24, 1·49)* |
| 24 to <48hr | 2·87 (2·33, 3·55)* | 2·89 (2·34, 3·58)* | 2·89 (2·32, 3·57)* |
| ≥48hr | 4·24 (3·58, 5.03)* | 4·37 (3·69, 5·18)* | 4·26 (3·59, 5·05)* |
| Never Breastfed | 4·94 (3·65, 6·69)* | 5·23 (3·86, 7·08)* | 6·12 (4·40, 8·47)* |
| * p<0.001 | | | |
| ^a^ Excluding children (n=6340) whose mother reported maternal complications (prolonged labour or fever) during the time of delivery | | | |
| ^b^ Excluding children (n=470) who died in the first 48 hours | | | |
| ^†^ Adjusted for sex of child, birthweight, instrument boiled before the cord was cut, application of material after cutting cord, time of first bath, timing of drying, colostrum, gestational age at birth, parity, type, place and skilled attendance at delivery, stillbirth/miscarriage of previous child, prolonged labour during childbirth, fever (mother) at childbirth, assets and the treatment arms. Also adjusted for clustering of participants from 100 clusters in five districts using multilevel random effects (clusters nested within district) | | | |
| ^††^ Adjusted for all the above-mentioned variables except- prolonged labour during childbirth and fever (mother) at childbirth. Also adjusted for clustering effect | | | |
| ^c^ All potential covariates adjusted for in these regression models are continuous variables | | | |
